# Importance of Participation in Major Life Areas Matters for Return to Work

**DOI:** 10.1007/s10926-014-9545-2

**Published:** 2014-10-16

**Authors:** Lisbeth Kvam, Kjersti Vik, Arne Henning Eide

**Affiliations:** 1 Department of Occupational Therapy, Faculty of Health and Social Work, Sør-Trøndelag University College, Trondheim, Norway; 2 Sintef, Technology and Society, Oslo, Norway

**Keywords:** Vocational rehabilitation, Participation, Gender differences, Return-to-work, Longitudinal study

## Abstract

*Introduction* The complexity of the process and outcome of vocational rehabilitation yearns for a multifaceted approach. This article investigates whether importance of participation in major life areas for men and women predicts the outcome of vocational rehabilitation. *Methods* This longitudinal study provides measure points at the start of the intervention (T1), at the end of the intervention (T2) and at a follow-up 6–12 months after completing the rehabilitation program (T3). Associations were assessed by nominal logistic regression. *Results* The importance of participation in work was positively associated to return to work (RTW), while the importance of participation in leisure activities and importance of participation in family was negatively associated with RTW after the rehabilitation. Gender and number of children also contributed significantly to the regression model. *Conclusion* To identify individuals’ subjective evaluation of the importance of participation may be of value in explaining return or not RTW and contribute to explain gender differences in outcomes. It may also inform rehabilitation counselors in collaboration with clients and facilitate tailoring interventions to the individual’s needs.

## Introduction

Existing evidence on the impact of vocational rehabilitation on return to work (RTW) for men and women with musculoskeletal pain is diverse. The complexity of the rehabilitation process and in predicting outcome is widely agreed. The wide range of factors associated with the outcome of vocational rehabilitation include demographic, psychological, social, medical, rehabilitation characteristics, work-place characteristics, factors related to the social security system, and general unemployment [1].

Several studies have found that men more often RTW after vocational rehabilitation than women [2, 3]. Previous research has suggested that RTW subsequent to rehabilitation, and particularly gender differences, should be assessed by a multifaceted approach that can interface with the complex and non-linear relationship between work participation, health, and socially related issues [4–6]. Such an approach is represented by the International Classification of Functioning, Disability and Health (ICF) [7], which is a bio-psycho-social model that conceptualizes health and illness. The model provides a starting point for investigating factors that influence the outcome of vocational rehabilitation. Research have shown that the ICF has contributed to the understanding and development of systematic schemes of the multitude of factors that matter in vocational rehabilitation, as well as shedding light on interactive and evolutionary aspects that influence the process and outcome [8]. The ICF is comprised of four interrelated domains: body, structures, body functions, participation, activity, and environmental factors. Participation, defined as involvement in a life situation, is an important component of the ICF. The concept has attracted growing interest within rehabilitation research due to the possibility to capture components that relate to personal actions in a real-world environment [9]. Thus, understanding participation and change in participation in important life areas may contribute to explain outcomes of rehabilitation processes.

It has been argued that one has to take into consideration what matters to the individuals to be able to assess the problems in an useful way, be it in therapy or research [10]. Studies exploring the subjective meaning and experience of participation have found that participation is closely connected to context and values [11–13]. Norms and values in society may influence what men and women find most important. However, the centrality of work in contemporary western society, the responsibility to provide and maintain daily care for children and the responsibility to act in ways that promote good health, are all value driven aspects that may conflict [14]. It has been shown that men and women in vocational rehabilitation in some ways differ in their evaluation of the importance of participation in major life areas [15] and that different discourses of participation in work exists among men and women that are undergoing rehabilitation [16]. These findings suggests that subjectively perceived importance of participation in major life areas may be predicative of the outcome of rehabilitation through revealing the motivation for participation in work and other central life areas. Consistent with this view is the finding that the importance of work and the consequences of not working within a person’s context of life have been found to influence RTW [17]. In the same line, it has been suggested that participation restrictions at work are related to participation restrictions in other life domains [18]. However, because of the difficulties clarifying the construct of participation few have attempted to measure it [19].

In many studies individual’s own expectations of recovery and outcome of vocational rehabilitation have been shown to be a stable and important predictor of RTW [20–22]. Within Bandura’s social cognitive theory, self-efficacy is a key concept that is defined as a personal belief of how successfully one can cope with different situations [23]. Studies have linked self-efficacy to RTW suggesting that high self-efficacy promotes RTW while low self- efficacy hinders RTW [24–26]. Within the same paradigm, health locus of control refers to the degree of control that people believe they possess over their personal health [27]. Factors associated to health locus of control, such as fear-avoidance, subjective health complaints and illness perception [28, 29], and expectations of change in overall health during the course of rehabilitation [30], are by some authors found to influence RTW. Self-efficacy, health locus of control and other dimensions of core self-evaluations have been suggested to influence men and women differently [31]. The psychological factors related to believes and expectations are important for understanding RTW subsequent to vocational rehabilitation. However, it remains unclear if self-efficacy and health locus of control is affecting RTW or if contextual aspects related to the course of the rehabilitation process and a person’s life situation influence believes and expectations [32, 33]. A knowledge gap has been identified, concerning the meaning and formation of expectations, and the process that workers go through when determining their RTW goals [34]. Approaching the issue from a bio-psycho-social perspective, captured in the concept of participation, implies a shift from handicap and personal traits to a stronger emphasis on factors enhancing participation and less emphasis on negative emotions and depression [35]. Considering findings that culture impacts on self-efficacy [36] and that taking contextual variables, such as class and gender, into health locus of control, indicates that individuals’ sense of control may be attributed to their relative chances of control and influence [37]. In a previous study we found that expectations of participation in major domains of life among men and women with musculoskeletal pain in vocational rehabilitation was goal-oriented and affected by personal (e.g. gender), environmental and/or societal factors (e.g. family situation, attitudes of others) and physical and psychological aspects, which most likely influence the rehabilitation process and outcome [38]. While it is expected that environmental, medical and personal issues are associated with RTW after vocational rehabilitation, to our knowledge, no study has investigated the relationship between importance of participation in major life areas and the outcome of vocational rehabilitation. We believe that doing this may add to the explanation of the complex processes associated with the outcome of vocational rehabilitation and particularly expand the knowledge considering gender differences.

## Research Aim

This study aims to illuminate whether men and women’s evaluation of importance of participation in major life areas predicts RTW following vocational rehabilitation.

## Materials and Methods

### Design

This study was longitudinal with measure points at the start of the intervention (T1), at the end of the intervention (T2) and at a follow-up 6–12 months after completing the rehabilitation program (T3). Approval was obtained from the Norwegian Social Science Data Service (NSD).

### Participants and Study Context

A total of 270 individuals participated; 191 were women and 79 were men, aged 21–64 (mean age 43.8 years, SD = 10.2). The majority, 77 % (*N* = 168), had upper secondary education and training while 23 % (*N* = 51) had higher education. For further characteristics of the sample see Table 1. The target group for participation in the rehabilitation program was people with complex health conditions such as musculoskeletal pain sometimes in combination with mild psychological problems. They were all enrolled in one of two vocational rehabilitation institutions, both in areas with a combination of rural and small town characteristics in Mid-Norway. All were undergoing multidisciplinary vocational rehabilitation from October 2010 to December 2011. The duration of the program was individual, ranging from 4 to 20 weeks with a majority of participants undergoing 12 weeks of rehabilitation 3 days per week on an out-patient basis. The participants were admitted to the rehabilitation program based on referrals from their general practitioners, social insurance officers and some on their own initiative.

**Table 1:** Characteristics of study participants (*N* = 270 at baseline)

	*N*	%
Women		
T1	191	70.7
T2	103	73.6
T3	138	73.8
Men		
T1	79	29.3
T2	37	26.4
T3	49	26.2
Employed at T1	166	61.5
Men employed <100 %	17	33.3
Women employed <100 %	69	60.0
Sick leave 100 % at T1	219	81.2
Men	64	83.3
Women	155	80.3
Sick leave <100 % at T1	27	10.0
Men	7	16.7
Women	20	19.7
Full RTW at T3	9	3.3
Men	9	9.9
Women	0	0.0
Partly RTW at T3	62	33.0
Men	16	20.3
Women	46	24.1
Full work assessment allowance at T3	73	27.0
Men	19	10.2
Women	54	28.9
Full disability pension at T3	29	10.7
Men	7	3.7
Women	22	11.8

The program was free of charge and participants were compensated for lost income by the Norwegian Labor and Welfare Service (NAV). All participants had an individual plan prepared in collaboration with rehabilitation staff at the start of the intervention. The program comprised of individual activities, group activities and work-related activities at the rehabilitation premises and at the workplace. Education in health- and work-related themes and adaptation at the workplace were implemented to enhance participation in work as outcome. The intervention aimed at changing the individual’s focus from pain and disability to recourses and competencies.

### Data Collection

A battery of self-report measures were routinely administered to the participants in the intervention at T1 (*N* = 270) and at T2 (*N* = 140). In the period from October 2010 to December 2011 an additional measure, comprised of participation-related questions, was integrated into the ordinary data collection. The follow-up survey was administered to the 270 participants by regular post 6–12 months after finishing rehabilitation. A total of 109 participants responded at first notice and the remainder were contacted by phone after 4–6 weeks and asked if they would answer the survey orally. This procedure ended in a response rate of 69 % (*N* = 187) at T3. There were no significant differences with regard to gender or age between the dropouts and those who completed the questionnaire at T1, T2 and T3 (Table 2).

**Table 2:** Correlations between the items in the outcome variable and the independent variables at T1 and T2 using Pearson’s correlation

	Work 100 %	Work <100 %	WAA 100 %	Disability pension 100 %
Age	−.009	.040	−.212***	.258***
Number of children	.019	.099	−.150**	.033
T1 overall health	−.055	−.153*	.168**	.161**
T2 overall health	−.117	−.170*	.180**	.152*
IPW T1	.138**	.290***	−.096	−.376***
IPW T2	.189**	.239***	−.090	−.319***
IPF T1	.019	−.045	.055	.079
IPF T2	.084	−.105	−.085	.173**
IPL T1	−.191***	.168**	−.084	.101*
IPL T2	−.050	−.064	−.032	.132*

*** *p* < .001, ** *p* < .050, * *p* < .10

### Measurements

Among the self-report measures was the Norwegian version of the Nottingham Health Profile, the Medical Outcomes Study 36-item Short-Form Health Survey COOP/WONCA Charts measuring health perception [39]. This scale consists of six charts and is based on self-reporting on the general state of health and functioning during the past 2 weeks. Charts 1–6 represent the different domains of physical fitness, emotional well-being, daily activities, social activities, changes in health and overall health. The charts are rated from 1 (good functional status) to 5 (poor functional status). The scale has demonstrated acceptable levels of construct validity, test–retest reliability and sensitivity to change [39]. The COOP/WONCA assessment was chosen due to previous findings indicating that health perception is partly predictive of RTW after vocational rehabilitation [28]. Bivariate correlations between the items included in the outcome variable and the charts of COOP/WONCA showed significant correlations only for overall health. Thus, only the chart on “overall health,” which shows the participants’ ratings of their general health over the previous 4 weeks, were included in the analyses.

Researchers developed a measure that captured important issues affecting participation in work among the group studied. The participation questions were developed on the basis of findings of a previous qualitative study that investigated the experiences of participation among men and women conducting multidisciplinary vocational rehabilitation at the two rehabilitation sites [38]. Previous research on participation [11–13] was further used to provide theoretical and empirical directions.

To improve validity of the participation measurements at T1 and T2, a pilot-study was conducted with four women and two men who were recruited among individuals going through the vocational rehabilitation program in September 2010. Each of the participants in the pilot study answered the survey questions followed by a focus group discussion. The aim of the discussion was to identify questions, words, phrases, etc., that were not distinct, and to discuss ways to rewrite the questions to make them more intuitive. Second, the participants were asked to discuss if the questions were meaningful to them considering the context and process of vocational rehabilitation. Third, the discussion revolved around whether questions or themes were lacking. The pilot-study indicated an approval of findings of a previous study that work, family life and leisure time covered the most important domains of life for this group [38]. While the pilot-study led to changes in structure and wording, the content of the questionnaire remained largely unchanged.

After evaluating the results from the pilot study the participation measure consisted of 55 items divided into six subscales: 1) importance of participation in work, family life and leisure activities at the measure point, 2) importance of participation in work, family life and leisure activities before sick leave, 3) perceived barriers for RTW, 4) perceived issues with regard to the importance of having a job, 5) expectations of work participation in the future and 6) issues concerning perceived optimal participation in general. The internal consistency of the whole instrument was good with a Cronbach’s alpha coefficient of 86 [40].

In the current study the items in subscale 1 were used as independent variables. The items of the subscale were importance of participation in work (IPW), importance of participation in family (IPF) and importance of participation in leisure activities (IPL) at measure point. Responses were captured by a Likert scale ranging from 1(participation is not important at all) to 7 (participation is very important) and measured at T1 and T2. The internal consistency of subscale 1 was acceptable with a Cronbach’s alpha coefficient of 73 [40].

Change in IPW, family and leisure activity variables were computed by subtracting level of importance at T1 from T2.

The follow-up survey consisted of 13 questions considering the situation at T3. The variables full-time work, part-time work, full work assessment allowance (WAA) and full disability pension were computed into a new variable scoring 1 for full-time work, 2 for part-time work and 3 for full WAA and full disability pension. The rationale for the construction of this outcome variable was to include individuals who selected only one of the response alternatives, while, at the same time, capturing some nuances of outcome alternatives by differentiating between full RTW and partial RTW.

### Statistics

SPSS version 20.0 (IBM Corp., Armonk, NY, 2011) for Windows was used for the statistical analyses. Initially, frequencies were examined and bivariate associations estimated. Paired sampled *t* tests were conducted to investigate prospective changes in importance of participation and changes in perceived overall health from T1 to T2; the analysis were conducted for all and for men and women separately. The results from the bivariate analyses (Chi square tests, Pearson’s correlation and bivariate regression), together with theoretical and empirical findings from previous research, guided the decision of which independent variables to include in the final regression model. Independent variables examined were IPW, IPF, IPL, change in IPW, change in IPF, change in IPL, gender, number of children, age, overall health, and education.

Because the outcome measure in this study was a variable with three categories, multinominal logistic regression was performed. The reference category was full long term benefits (WAA and disability pension). To estimate the odds of membership to the outcome group, odds ratios (OR) and their 95 % confidence intervals (95 % CI) were used. Multivariate logistic regression was finally performed with all retained independent variables in the final regression model.

## Results

A Chi square test for independence indicated a significant association between gender and working full-time after the rehabilitation, χ^2^ (1, *N* = 187) = 13.01, *p* = .001. No significant associations were found between gender, part-time work, WAA and disability pension. With regard to education the Chi square test showed no association to either of the categories of the outcome variable and education was, therefore, excluded from further analysis. The “change in participation” variables did not correlate to any of the other variables nor significantly contribute in the bivariate regressions. Neither did the paired sample *t* test comparing T1 and T2 yield any significant mean change in the importance of participation variables, a finding also applicable when running the analyses by gender. Therefore, the “change in participation” variables were excluded from further analysis.

The variable, participation in family, had the least correlation to the items in the outcome variable (positively correlated to disability pension at T2); however, at T1 it was also positively correlated with the number of children (r = .21, *p* = .001) and negatively correlated to gender (r = −14, *p* = .024). Previous research has indicated that importance of family might for women indicate being present for children while for men importance of family is more linked to providing for children [15], and therefore we chose to include importance of family as a variable in the multivariate regression model.

The paired sample *t* test did yield significant positive change in perceived overall health from T1 (M = 3.31, SD = .84) to T2 (M = 3.04, SD = .86), *t* (134) = 3.30, *p* < .005 (two-tailed). The eta square statistic (.07) indicated a moderate effect size. When running the analyses by gender, we found that the positive change in perceived overall health applied to women from T1 (M = 3.26, SD = .82) to T2 (M = 2.97, SD = .85), *t* (99) = 2.97, *p* < .005 (two-tailed). The eta squared statistic (.08) indicated a moderate effect size. No significant change was found for men.

In bivariate regression analyses the variables IPW, IPF, IPL, overall health and gender were statistically associated to the outcome variable. Individuals who demonstrated a higher evaluation of participation in work were more likely to be found in “Full RTW” (OR: 2.48, 95 % CI: 1.34–4.60) or in the “Partly RTW” (1.44, 95 % CI: 1.20–1.73) categories. Those reporting low IPL were more likely to be found in the “Full RTW” (OR: .43, 95 % CI: .25–.74) category. With regard to gender men were far more likely than women to be found in the “Full RTW” (OR: .09, 95 % CI .02–.48) category (Table 3).

**Table 3:** Bivariate multinominal logistic regression of baseline importance of participation variables predicting full RTW (*N* = 9) versus full benefit (*N* = 99) and partly RTW (*N* = 62) versus full benefit 6–12 months subsequent to vocational rehabilitation

	OR	(95 % CI)
Full RTW versus full benefit		
IPW	2.48	(1.34–4.60)*
IPF	1.42	(0.60–3.37)
IPL	0.43	(0.25–0.74)*
Gender	0.09	(0.02–0.48)*
Number of children	1.29	(0.70–2.35)
Age	0.99	(0.93–1.06)
Overall health	0.42	(0.17–1.08)
Partly RTW versus full benefit		
IPW	1.44	(1.20–1.73)*
IPF	0.79	(0.57–1.11)
IPL	1.04	(0.82–1.31)
Gender	0.95	(0.45–1.97)
Number of children	0.20	(0.91–1.55)
Age	1.00	(0.97–1.03)
Overall health	0.54	(0.36–0.83)*

Unadjusted odds ratios (OR) and 95 % confidence intervals are shown

The variable gender; women were reference category

The variable age was continuous ranging from 21 to 64

* *p* < .05

In the multivariate analysis IPW remained statistically associated with both “Full RTW” and “Partly RTW” (OR: 3.69, 95 % CI 1.10–12.56) and (OR: 1.42, 95 % CI 1.16–1.73) respectively. Consequently, to evaluate participation in work highly remains equally important for RTW when bringing the other variables into the equation. Also IPL is significantly contributing to the multivariate model. Individuals reporting lower evaluation of IPL (OR: .16, 95 % CI .03–.98) were more likely to RTW full time. Likewise gender remained statistically associated with “full time RTW” (OR: .01, CI 5.699E −005–.51) showing that men were much more likely than women to be found in the “full time RTW” category. In this sample 9 men and none of the women returned to full time work. Due to the relatively few persons in the “Full time RTW” group the confidence intervals around the estimated odds ratio were very wide. In summary, when comparing the likeliness to belong to the “Full RTW” category or the “Full benefit” category 6–12 months after rehabilitation, the model indicates that being male and evaluating IPW highly and IPL as less important increases the probability of a full RTW. None of the other variables contributed to the model. When comparing the likeliness to be found in the “Partly RTW” group or the “Full benefit” group the results in the multivariate analysis differ from the bivariate analysis. First, we see that IPF becomes statistically associated (OR: 0.67, 95 % CI 0.45–1.00) with the “Partly RTW” category. Low evaluation of the IPF slightly increases the likeliness for RTW in a part time position. In the opposite direction having (more) children increases the likeliness (OR: 1.44, 95 % CI 1.04–2.00) to be found in the partly RTW category. Gender, IPL, age and overall health was not statistically associated to the outcome categories when comparing “Partly RTW” and “Full benefit” 6–12 months subsequent to rehabilitation (Table 4).

**Table 4:** Multivariat multinominal logistic regression (full factorial) of baseline variables predicting full RTW (*N* = 9) versus full benefit (*N* = 99) and partly RTW (*N* = 62) versus full benefit 6–12 months subsequent to vocational rehabilitation

	OR	95 % CI
Full RTW versus full benefit		
IPW	3.69	(1.10–12.56)*
IPF	3.95	(0.44–35.57)
IPL	0.16	(0.03–0.98)*
Gender	0.01	(5.699E −005–0.51)*
Number of children	3.63	(0.93–14.23)
Age	0.87	(0.68–1.12)
Overall health	0.21	(0.04–1.28)
Partly RTW versus full benefit		
IPW	1.42	(1.16–1.73)*
IPF	0.67	(0.45–1.00)*
IPL	1.04	(0.81–1.34)
Gender	0.88	(0.35–2.23)
Number of children	1.44	(1.04–2.00)*
Age	1.00	(0.96–1.05)
Overall health	0.61	(0.36–1.04)

R^2^ = .38 (Cox & Snell), .42 (Nagelkerke). Model χ^2^ (58.746), *p* < .001

The variable gender; women were reference category

The variable age was continuous ranging from 21 to 64

Adjusted odds ratios (OR) and 95 % confidence intervals are shown

* *p* < .05

## Discussion

This study aimed to investigate simultaneously the role of importance of participation in major life areas and the variables gender, number of children, age and overall health to explain the outcome 6–12 months after conducting vocational rehabilitation.

The rehabilitation intervention did not seem to have an impact on the individual’s evaluation of the IPW as IPW was stable during rehabilitation, and showed strong association to RTW fully and partly. It is possible that the rehabilitation reinforced the individual’s goal orientation toward RTW for those already perceiving work as important, while the opposite might apply to those who did rate participation in work as less important. Both scenarios are relevant from the rehabilitation professional’s point of view; it is of interest to reflect on which working mechanisms push some toward work disability and others toward RTW. To see participation in work as important most likely strengthens the motivation to RTW. Indeed, motivation is argued to be an important predictor of rehabilitation outcomes [41, 42] as is change in work motivation [43], and to be highly motivated is related to having a goal of RTW [44]. Likewise, self-efficacy expectations have been found to be predictors of RTW [45] and may be a component of the explanation. It must be taken into account that most of the participants in the rehabilitation program had a long history of health problems. To be referred to vocational rehabilitation was for the majority a final trial to RTW after been subject to extensive medical treatment [5]. Consequently, the perceptions regarding IPW and other major life areas had been developed for some time prior to starting vocational rehabilitation. This finding, seen together with the strong correlation between IPW and RTW both partly and fully, may indicate that measuring IPW before starting the intervention is of value for individual shaping of the rehabilitation process. Hence, one might suspect that a person who does not view participation in work as important has different needs and a more complicated route to RTW than a person who believes participation in work is very important.

Health problems are among the factors which have been found to significantly predict the outcome of vocational rehabilitation [46]. However, other studies have found medical aspects less important [22]. The results in the current study showed that poor perceived overall health correlated to receiving long-term benefits, while better perceived overall health correlated to a partial RTW; however, the health variable did not significantly predict the outcome in the regression model. The results do not rule out that the perception of overall health influences RTW, thus IPW and individual’s perception of their health might be interactive components from the start. For instance, studies investigating participation have shown how people with disabilities participate does not necessarily reflect their ability to participate or perform activities, but most likely relates to the perception of their own possibilities, which is influenced by health, family, economy, job-market situation, etc. [47]. The same may be applicable for self-efficacy expectations and health locus of control variables [36, 37]. In the current study reported overall health improved significantly during the rehabilitation for women only. Yet, in this sample men were far more likely than women to RTW full-time, while no gender difference was found regarding partial RTW. This may indicate that importance of participation comes about in interaction with other factors. An interruptive factor, which may have influenced the findings, was that the women were more likely to work part-time in the first place. Consequently, their incentive to RTW full-time was probably weaker than for most of the men, a circumstance that could also affect self-efficacy believes. One can ask if women’s lower work participation reflect the fact that combining full-time work and participation in the family was challenging from the start. If this was the case, it underlines a gendered mechanism that probably was enhanced by the entry of long lasting health problems.

Those who had children were more prone to RTW partly in the current study. When providing for children, the economic incentive to work is obvious. However, we did not control for the children’s ages. A dimension of the effect of having children on partial RTW might be that individuals still providing for children are more inclined to go back to work compared to those having older children or who do not have children at all. Returning to work part-time instead of full-time can also be a result of wanting to be present for the children, hence, a combination of providing for and being present for children. On the other hand, to see participation in family as important reduced the odds of returning to work part-time compared to receiving full benefits. A recent study on sickness absence found that the risk for work absenteeism is higher among women with children than without children. The risk decreased by age and was not found after the age of 35, except for single mothers who had a higher risk irrespective of age [48]. The effect of parenting on reduced participation in work was not found for men [49]. In qualitative studies indications of gender differences in the effect of family and children on RTW have been discussed [6, 38]. Previous research has indicated that women contrary to men perceive living with children as a hindrance for RTW when they start rehabilitation [15]. Albeit these findings point to a possible gender effect regarding motivation to RTW partly when having children, these studies do not fully explain the results in the current study. We suggest that further research including more detailed participation items as well as taking into account the age of the children, and conducting the regression analyses for men and women separately could illuminate different dimensions of gender issues with regard to the influence of family and children on RTW.

In the current study age did not predict either full or partial RTW, a finding which is not in line with the majority of other studies [17, 46, 50, 51]. As shown in Table 2, older age was related to the granting of full disability pension, while younger age was correlated to receiving full WAA 6–12 months after rehabilitation. WAA is a long-term benefit which is granted to individuals who are typically younger and unclear as to workability, albeit still regarded as candidates for improvement of health with a subsequent RTW [52]. Thus, the indication that age is correlated to disability pension and WAA is logical. Still, the results in this study indicated that age seemed less important in predicting the outcome of the rehabilitation. Hence, the results imply that age most likely interacts with other aspects in a person’s life and is a component of a mechanism rather than a sole predictor for RTW.

Low importance of leisure activities was associated to a full RTW, but was not predictive of a partial RTW. A simple explanation may be that some individuals struggling with prolonged health problems cannot manage both full-time work and being active in their spare time. A study investigating vocational and leisure outcomes subsequent to spinal cord injuries found that loss of participation in work tended to be replaced by domestic and leisure activities [53]. When prolonged work disability occurs, leisure activities may be important to maintain and find new meaning in everyday life [54]. In addition, focusing on leisure activities may be a way to uphold health and contribute to the community [38]. In a gender perspective it is worth to mention that only men were found in the “Full RTW” category and for these men leisure activities may be less relevant because participation in work is perceived more important than other life areas, a finding that corresponds with gender role theory [55]. Bivariate correlation analyses showed that IPL correlated strongly negative to full RTW, and had a week negative correlation partly RTW and full disability pension. This may indicate that participation in leisure activities have some underlying effect on the outcome of vocational rehabilitation, however, most likely in interaction with variables or circumstances that this study’s regression model did not capture.

A characteristic of the results in this study is that the significant associations from the bivariate analyses remained stable when conducting multivariate analysis were the variables IPF and number of children also became statistically associated to the outcome. This may underline the interactive aspect of factors playing a role for RTW and indicate that considering multiple aspects is required when aiming at understanding and explaining mechanisms in work in the process and outcome of vocational rehabilitation.

## Study Limitations

A common limitation to logistic regression analyses is that the estimates are affected by omitted variables, even when these variables are unrelated to the independent variables in the model. Thus, the effects reflect unobserved heterogeneity, and it is, therefore, problematic to interpret OR as substantive effects [56]. In the current study we did not have other similar quantitative studies to use as a basis for including variables. Consequently, our approach must be seen as exploratory, and an attempt to open the “black box” of importance of participation’s influence, and to determine if measuring importance of participation is meaningful and adds new knowledge, rather than claiming to explain cause and effect with regard to the outcome of vocational rehabilitation. As suggested by Mood (2010), we carefully examined the variables, statistically and qualitatively, at both T1 and T2, considering them for inclusion in the multivariate regression model. As a result the variables IPF and “number of children” was retained even though they correlated to only one of the response alternatives in the outcome variable (see Table 2). The size of the sample was not large enough to expand the regression model further with all variables that could possibly be associated with the dependent variable. We do, however, argue that the analyses performed and the variables used are suited to meet the scope of this article. There was a relatively large amount of missing answers from T1 to T2 which may have lowered the reliability of the results. However, a mitigating factor was that the missing answers were highly random.

Another possible limitation was the construction of the outcome variable because although WAA and disability pensions are long time benefits they are intended for individuals with RTW prospects and individuals who have no chance of RTW, respectively. However, we argue that the merging of the two categories were appropriate considering the purpose, which was to study specifically individuals who were not working at all at T3.

## Conclusion

The finding that IPW, family and leisure activities, together with other factors, contributed to the prediction of RTW, illuminates how attention to everyday life and what matters to people can help to explain RTW and failure to RTW. For rehabilitation the results point to a need for identifying the individual’s subjective evaluation of importance of participation in different life domains, in order to tailor the rehabilitation to each person to optimize RTW. The attempt to capture perceived importance of participation in different life domains does, in our view, contribute to the development and conceptualizing of the application of a bio-psycho-social perspective in RTW research. Thus, it delineates how RTW, which is the goal of vocational rehabilitation, is reciprocally influenced by health conditions, environmental factors and personal factors. Consequently, the approach may be useful in capturing issues that are difficult to explain, such as gender differences in outcome of vocational rehabilitation. Finally this study has indicated that measuring importance of participation may be of value both to explain RTW or not, and to rehabilitation staff as a helpful tool in the collaboration with clients. There is, however, a need to develop more nuanced participation instruments that better capture the broad specter of participation, and which also ensure the value, context and situational aspects to enable clinical and scientific use of the subjective evaluation of importance of participation. In order to increase clinical utility of the research, a deeper knowledge regarding the interactive aspects of the domains comprised in the ICF is needed. Thus, and based on the current body of knowledge, it is of interest to include self-efficacy variables together with participation variables in statistical models to expand on the knowledge of the formation of expectations of RTW in a truly bio-psycho-social and interdisciplinary perspective.
